# Turbulent Flow Over Large Roughness Elements: Effect of Frontal and Plan Solidity on Turbulence Statistics and Structure

**DOI:** 10.1007/s10546-017-0317-3

**Published:** 2017-11-04

**Authors:** M. Placidi, B. Ganapathisubramani

**Affiliations:** 1 City, University of London, London, EC1V 0HB UK; 2 University of Southampton, Southampton, SO17 1BJ UK

**Keywords:** Turbulent boundary layer, Diagnostic plot, Proper orthogonal decomposition, Wind-tunnel experiments, Urban roughness, Outer-layer similarity

## Abstract

Wind-tunnel experiments were carried out on fully-rough boundary layers with large roughness ($\delta /h \approx 10$, where *h* is the height of the roughness elements and $\delta$ is the boundary-layer thickness). Twelve different surface conditions were created by using LEGO™ bricks of uniform height. Six cases are tested for a fixed plan solidity ($\lambda _\mathrm{P}$) with variations in frontal density ($\lambda _\mathrm{F}$), while the other six cases have varying $\lambda _\mathrm{P}$ for fixed $\lambda _\mathrm{F}$. Particle image velocimetry and floating-element drag-balance measurements were performed. The current results complement those contained in Placidi and Ganapathisubramani (J Fluid Mech 782:541–566, 2015), extending the previous analysis to the turbulence statistics and spatial structure. Results indicate that mean velocity profiles in defect form agree with Townsend’s similarity hypothesis with varying $\lambda _\mathrm{F}$, however, the agreement is worse for cases with varying $\lambda _\mathrm{P}$. The streamwise and wall-normal turbulent stresses, as well as the Reynolds shear stresses, show a lack of similarity across most examined cases. This suggests that the critical height of the roughness for which outer-layer similarity holds depends not only on the height of the roughness, but also on the local wall morphology. A new criterion based on shelter solidity, defined as the sheltered plan area per unit wall-parallel area, which is similar to the ‘effective shelter area’ in Raupach and Shaw (Boundary-Layer Meteorol 22:79–90, 1982), is found to capture the departure of the turbulence statistics from outer-layer similarity. Despite this lack of similarity reported in the turbulence statistics, proper orthogonal decomposition analysis, as well as two-point spatial correlations, show that some form of universal flow structure is present, as all cases exhibit virtually identical proper orthogonal decomposition mode shapes and correlation fields. Finally, reduced models based on proper orthogonal decomposition reveal that the small scales of the turbulence play a significant role in assessing outer-layer similarity.

## Introduction and Background

Townsend (1976) first introduced the concept of outer-layer similarity, which states that the structure of the turbulence, if appropriately scaled, is unaffected by the surface roughness at a sufficient distance from the wall. This hypothesis is valid provided that the Reynolds number is sufficiently high ($hU_{\tau }/\nu>>1$), and that the height of the roughness is small compared with the boundary-layer thickness, $h<<\delta$. Here, $U_\tau$ is the skin-friction velocity, *h* is the height of the roughness elements, $\delta$ is the boundary-layer thickness, and $\nu$ is the kinematic viscosity of the fluid. Outer-layer similarity implies that turbulent motions and their characteristic structures are independent of the wall topology, and roughness acts merely to increase the surface stress without causing structural changes in the flow (Raupach 1992). The first studies to offer experimental support of outer-layer similarity include Perry and Abell (1977), Andreopoulos and Bradshaw (1981) and Acharya et al. (1986), who found good agreement between scaled mean velocity-defect profiles in the outer layer over both smooth and rough walls. Further support to outer-layer similarity in both mean velocity profiles and turbulence quantities can be found in, e.g., Perry and Li (1990), Raupach (1992), Schultz and Flack (2005), Wu and Christensen (2007), Volino et al. (2007), Castro (2007), Wu and Christensen (2010), Amir and Castro (2011).

However, some researchers have questioned the validity of outer-layer similarity, stating that roughness effects can be observed well into the outer layer (Krogstad and Antonia 1999; Keirsbulck et al. 2002; Tachie et al. 2004; Volino et al. 2009, 2011; Lee et al. 2010). An important factor leading to violation of outer-layer similarity also appears to be the three-dimensionality of the roughness morphology. Keirsbulck et al. (2002), Volino et al. (2009, 2011) all reported differences in the Reynolds stresses for two-dimensional transverse bars when compared with smooth walls. Krogstad et al. (1992) also reported a lack of outer-layer similarity on mesh-screen rough walls, where the roughness was found to significantly increase the wall-normal fluctuating velocity component and the second and fourth quadrant contributions to the Reynolds shear stress.

Jimenez (2004) reviewed most of the studies on rough-wall boundary layers and suggested that the agreement or violation of Townsend’s similarity hypothesis depends on the relative height of the roughness, $\delta /h$. Violation of outer-layer similarity appears for ‘strong roughness’ ($\delta /h<40$), meaning that the typical roughness-element height exceeds a few percent of the boundary-layer thickness. Others also drew similar conclusions, suggesting slightly different values for the critical $\delta /h$. Ligrani and Moffat (1986) stated that the breakdown of outer-layer similarity is to be expected when the extent of the roughness sublayer is larger than the inner layer itself. Flack et al. (2005) argued that the important parameter for the validity of outer-layer similarity is the ratio between the boundary-layer thickness to the equivalent sand-grain roughness height $h_s$ (Nikuradse 1933), and suggested a critical value of $\delta /h_s\ge 40$ for outer-layer similarity to be valid. Wu and Christensen (2007) noted wall similarity in both mean velocity profiles and Reynolds stresses for $\delta /h=28$ ($<40$) and $\delta /h_s=48$ ($>40$), offering further support to the importance of the equivalent sand-grain roughness height formulated by Flack et al. (2005). It is important to point out that Mejia-Alvarez and Christensen (2013), in a later study on the same roughness morphology, found this surface to have a tendency to promote channelling of the flow in the form of low- and high-momentum pathways, which influence the structure of the turbulence, causing the persistence of spanwise heterogeneity across the entire boundary layer. These secondary flows are accentuated when the spacing of the roughness elements is roughly proportional to the boundary-layer thickness and do not appear for cases with finer spacing (Vanderwel and Ganapathisubramani 2015).

The rough surfaces comprised of cubes studied by Castro (2007) also represent an interesting case, as these flows conform to outer-layer similarity up to $\delta /h\approx 5$, corresponding to a situation in which the mean height of the roughness elements exceeds some 50$\%$ of the boundary-layer momentum thickness $\theta$. Amir and Castro (2011) further confirmed the validity of outer-layer similarity in the mean velocity profiles up to $\delta /h\approx 5$, and also reported an onset of $\delta /h \approx 6.7$ for the Reynolds stresses to conform to outer-layer similarity. Numerical simulations (Leonardi et al. 2003; Hagishima et al. 2009) over cubic roughness elements for different patterns have shown that outer-layer similarity may apply up to $\delta /h\approx 6-8$, which is in contrast with other studies with a much smaller roughness that apparently violate outer-layer similarity (Bhaganagar et al. 2004; Bakken et al. 2005).

The critical ratios introduced by Jimenez (2004), Flack et al. (2005) and others seek to find a useful roughness characterization able to predict a priori the drag generated by a wall, based purely on the geometric characteristics of the surface morphology, which has recently been emphasized in Zhu et al. (2016). Numerous approaches have been applied to this problem (Moody 1944; Macdonald 1998; Grimmond and Oke 1999; Millward-Hopkins et al. 2011, among others), but a universal solution is yet to be found, as recently pointed out in Hong et al. (2012), Yang and Meneveau (2015) and Yang et al. (2016). Despite this, dating back to Schlichting (1937), the roughness morphology has been often described by two density parameters: the frontal and plan solidities. The frontal solidity $\lambda _\mathrm{F}$ is defined as the total projected frontal area of the roughness elements per unit wall-parallel area, while the plan solidity $\lambda _\mathrm{P}$ is the ratio between the plan area and the unit wall-parallel area (see Fig. 1 in Placidi and Ganapathisubramani 2015). When using $\hbox {LEGO}^{\mathrm{TM }} \hbox { blocks}$ as roughness, the pins characterizing the top of the bricks must be included when calculating $\lambda _\mathrm{F}$, following previous convention (Benson 2005; Padhra 2010). The roughness is classified here via the four parameters $\lambda _\mathrm{F}$, $\lambda _P$, $\delta /h$ and $\delta /h_s$, which have been chosen to facilitate comparisons to previous work that use regular roughness elements, and especially that carried out with cubic roughness elements.

Most of the previous numerical and physical experiments that systematically explored the effect of surface morphology on the flow have in fact been carried out with cubic roughness elements (Cheng and Castro 2002; Coceal and Belcher 2004; Kanda et al. 2004; Cheng et al. 2007; Santiago et al. 2008; Hagishima et al. 2009; Leonardi and Castro 2010 among various others). However, given that the frontal and top faces of a cube are identical, $\lambda _\mathrm{F}\equiv \lambda _\mathrm{P}$ by definition, means that these two parameters are coupled so that the effect of each cannot be determined independently. However, to our knowledge, only cubic roughness studies have been able to push the bounds for the validity of outer-layer similarity down to $\delta /h<10$, which suggests that cubes are a remarkable case of regular roughness for which this similarity holds. Therefore, the upper bound for the breakdown in outer-layer similarity is not entirely clear. It is also unclear whether the geometry and pattern of the roughness elements are important when assessing the outer-layer similarity. To answer these questions, we performed a series of experiments by systematically varying $\lambda _\mathrm{F}$ at a fixed $\lambda _\mathrm{P}$ and vice versa. A total of twelve different configurations (from sparse to dense regimes) were tested with the aim of examining the effect of (non-cubical) surface morphology on the mean velocity, turbulence statistics and structure, and thereby addressing the validity of Townsend’s similarity hypothesis in the case of a strong roughness ($\delta /h\approx 10$).

Section 2 introduces the details of the experimental facility and the methods used, and Sects. 3.3 and 3.4 then present the results on the validity of Townsend’s similarity hypothesis for various mean and higher-order quantities across different surface roughness conditions. Based on this analysis, Sect. 3.5 introduces and discusses a new criterion to assess outer-layer similarity. Sections 3.6 and 3.7 present results on the spatial structure of the flow obtained via proper orthogonal decomposition and the reduced-order models, respectively. Finally, Sect. 3.8 presents velocity correlation maps, with the final remarks and conclusions drawn in Sect. 4.

**Fig. 1:**
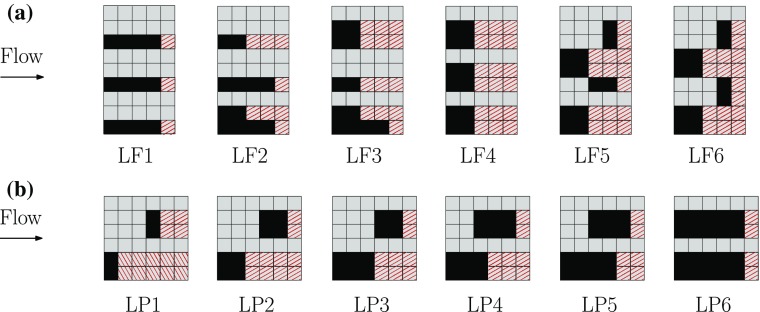
Roughness element patterns with **a** varying $\lambda _\mathrm{F}$ at $\lambda _\mathrm{P}=\mathrm{constant}=0.27$ and **b** varying $\lambda _\mathrm{P}$ at $\lambda _\mathrm{F}=\mathrm{constant}=0.15$. Dimensions are not shown to scale for ease of readability (basic square unit: 7.8 $\times$ 7.8 mm$^2$). The flow is from left to right. The shaded red areas are further discussed in Sect. 3.5. **a** Frontal solidity patterns, **b** plan solidity patterns

## Experimental Details

### Experimental Facility

The current experiments were carried out in the suction wind tunnel at the University of Southampton, whose adequacy for rough wall studies has previously been demonstrated (Reynolds and Castro 2008; Amir and Castro 2011). The tunnel working section is 4.5 m long and its cross-section measures 0.9 m $\times$ 0.6 m. The freestream turbulence intensity in the empty tunnel is homogenous and less than $0.3\%$. We refer to the streamwise, wall-normal and spanwise directions as (*x*, *y*, *z*) respectively. The plane $y=0$ is taken at the bottom surface of the baseboard onto which the roughness elements were located. The mean velocities along the three directions are denoted by the capital letters (*U*, *V*, *W*), while the corresponding fluctuating quantities are named $(u', v', w')$.

### Surface Roughness Morphology

LEGO™ baseboards in combination with differently arranged LEGO™ bricks were used to generate the different roughness configurations. The bricks have dimensions of 7.8 mm $\times$ 7.8 mm (streamwise $\times$ spanwise) and a fixed height of $h=11.4$ mm (including the pins at the top), and the geometry and patterns under examination are shown in Fig. 1. A total of twelve different patterns were tested to examine systematically the individual effects of frontal and plan solidities on the structure of the turbulence. The patterns denoted LF1 to LF6 represent cases for which $\lambda _\mathrm{F}$ is varied at a fixed $\lambda _\mathrm{P}=0.27$, whilst the plan solidity is varied in the cases denoted LP1 to LP6 at fixed $\lambda _\mathrm{F}=0.15$, as shown in Table 1. These different cases were positioned around the location of the peaks in surface drag based on previous studies (Grimmond and Oke 1999; Jimenez 2004).

**Table 1:** Experimental parameters for the variation in frontal and plan solidity

Case	$\lambda _\mathrm{F}$	$\lambda _\mathrm{P}$	$U_e$	$U_{\tau }$	*d* / *h*	$Re_{\tau }$	$\delta /h$	$\delta ^*/h$	$\delta /h_s$	${y_{0}}^{+}$
LF1	0.09	0.27	11.6	0.65	0.98	5110	10	1.67	69	10
LF2	0.12	0.27	11.6	0.73	0.59	6313	11	2.37	31	28
LF3	0.15	0.27	11.7	0.71	0.80	6140	11	2.28	36	24
LF4	0.18	0.27	11.5	0.80	0.75	6919	11	2.46	16	61
LF5	0.21	0.27	11.6	0.82	0.61	7092	11	2.81	13	77
LF6	0.24	0.27	11.6	0.81	0.73	7005	11	2.72	14	69
LP1	0.15	0.11	11.5	0.81	0.50	7642	12	3.07	12	89
LP2	0.15	0.22	11.6	0.78	0.68	6746	11	2.63	16	60
LP3	0.15	0.27	11.6	0.71	1.00	6140	11	2.46	30	29
LP4	0.15	0.33	11.5	0.67	0.91	5794	11	2.37	31	26
LP5	0.15	0.39	11.5	0.66	0.97	5189	10	2.11	39	18
LP6	0.15	0.55	11.4	0.67	0.97	5268	10	1.84	54	14

The aerodynamic parameters are calculated through a logarithmic-law fit with $\kappa =0.38$ in the range $1.5h\le y\le 0.2\delta$. The data presented are from the two-dimensional dataset; $U_e$ and $U_\tau$ are reported in m s$^{-1}$

### Determination of Skin Friction and Aerodynamic Parameters

The drag generated by the different rough walls was directly measured via a floating-element drag balance based on the design of Krogstad and Efros (2010). The sensing element was placed approximately 4 m downstream along the test section, where the flow had developed over a distance of $20\delta$ to ensure the fully-developed conditions similar to Antonia and Luxton (1971) and Castro (2007). However, recent studies have highlighted that the flow regains equilibrium rapidly downstream of step changes in surface roughness (Yang 2016). This location was also selected for the particle image velocimetry (PIV) measurements discussed in Sect. 2.4. Further details on the drag-balance construction, accuracy and validation can be found in Efros (2011), Placidi and Ganapathisubramani (2015) and Placidi (2015), respectively, where we note here that this independent measurment of the skin friction is much more accurate and reliable than the commonly-used logarithmic-law fitting procedures (Acharya et al. 1986; Castro 2007; Segalini et al. 2013). The drag results are not included here as they were extensively discussed in Placidi and Ganapathisubramani (2015). With the measured value of $U_\tau$, a least-squares fitting procedure was used to evaluate the zero-plane displacement *d* and the roughness length $y_{0}$ by

1 $$\begin{aligned} U^{+}=\frac{1}{\kappa }\ln \left( \frac{y-d}{y_0}\right) , \end{aligned}$$

which is valid in the fully-rough regime; here, $\kappa$ is the von Kármán constant. Since a value of $d>h$ would be physically meaningless in accordance with its definition in Jackson (1981), we force the condition $d\le h$ as performed by Iyengar and Farell (2001). A logarithmic layer was assumed for $1.5h\le y\le 0.2\delta$ consistent with Schultz and Flack (2005). The fitting procedure was carried out with $\kappa =0.38$, with further details and the uncertainty inherent in this method discussed in Placidi and Ganapathisubramani (2015).

### Particle Image Velocimetry

Planar (two-dimensional) and stereoscopic (three-dimensional) PIV measurements were acquired for all cases at a freestream velocity $U_\infty =$ 11.5 m s$^{-1}$ at approximately 4 m downstream in the canopy field. For all cases, the flow was seeded with a vaporized glycol-water solution of particles (1 $\mu$m in diameter) and illuminated by a 0.7-mm thick laser sheet produced by a pulsed laser system (New Wave Nd: YAG) operating at 200 mJ. The time delay between laser pulses was adjusted, such that the bias error of the PIV velocity measurements is less than 1% of the full-scale velocity (given that the subpixel determination has an uncertainty of about ± 0.1 pixels—see Adrian and Westerweel 2011). The overall uncertainty in turbulence quantities was determined following Benedict and Gould (1996) to be less than 1, 8 and 10% for the mean velocity, turbulence intensities, and the shear stresses, respectively, in accordance with previous studies (Wu and Christensen 2007). For the two-dimensional case, streamwise wall-normal planes (*x*, *y*) were acquired at the spanwise centreline of the test section using an ImagerProLX camera (4870$\times$3246 pixel$^2$) equipped with Nikon 105 mm f/8 lenses. The sampling frequency (0.8 Hz) was set so that statistically uncorrelated measurements were acquired for each image pair. For each run, 2000 image pairs were acquired and processed with *DaVis 8.0* software. With the resulting resolution approximately 0.7 mm $\times$ 0.7 mm for all cases, vectors are spaced at half that distance (i.e. a 50% overlap). Light reflections limited the wall-normal extent of the field-of-view for the two-dimensional measurements to the flow outside the canopy layer ($h<y<1.3\delta$), but spanned approximately $1.8\delta$ in the streamwise direction.

Given the severity of the surface roughness ($\delta /h\approx 10$), spanwise flow heterogeneity could be present, particularly within the roughness sublayer. To investigate this feature and to validate the two-dimensional datasets, stereoscopic PIV measurements were also carried out in the spanwise wall-normal plane (*y*, *z*) at the same location of the planar measurements. Two cameras with the same specifications as the two-dimensional measurements were used to acquire 1500 digital image pairs. Three-dimensional measurements were carried out for only six cases: LF2, LF3, LF5 and LP2, LP4, LP5 as these represent conditions for sparse, medium and dense regimes: refer to Placidi and Ganapathisubramani (2015) for further details. The results presented in the following sections have been filtered to match the local resolution as discussed in the Appendix. However, the filtered data has still a resolution comparable with previous cross-wire and PIV-based measurements reported in the literature.

### Smooth-Wall Dataset

Although primarily aimed at comparing different types of rough morphologies, results for a smooth-wall case are also briefly discussed for the sake of completeness. Given the importance of matching $Re_\tau$ across the rough and the smooth cases, smooth-wall data from Hutchins et al. (2009), at $Re_\tau \approx 7300$, are used for the comparison.

## Results and Discussion

### Assessing the Flow Homogeneity

**Fig. 2:**
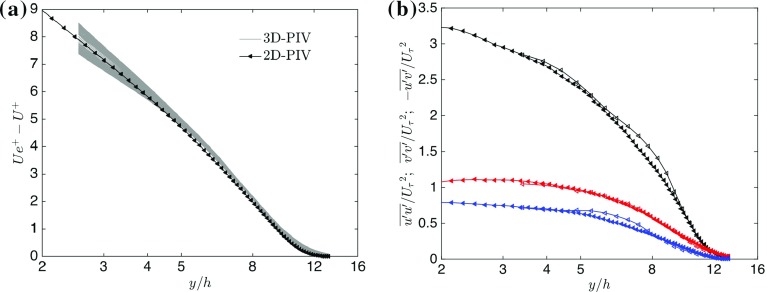
**a** Comparison of mean velocity-defect profiles across two-dimensional (black) and three-dimensional PIV (grey) measurements. Three-dimensional PIV data are reported at each spanwise location. **b** Flow homogeneity and validation between two-dimensional (full marks) and three-dimensional (empty marks) PIV data. The wall-normal variation of streamwise (black), wall-normal (red) turbulence intensity and Reynolds shear stress (blue). Both two- and three-dimensional data have been averaged across the field-of-view. Only one in every five vectors is plotted for clarity

It is important in these high relative roughness flows to investigate the variability of the statistics across the spanwise direction for which Fig. 2 compares two- and three-dimensional PIV results. Although only a particular case is presented for the sake of brevity, the conclusions drawn are valid for all cases. Figure 2a shows the profiles across the different spanwise locations (in grey), where, despite some flow heterogeneity in the roughness sublayer, very little difference is found in the mean streamwise velocity component across the spanwise locations in accordance with the criterion highlighted in Vanderwel and Ganapathisubramani (2015). Velocity profiles obtained via two-dimensional PIV (in black) are found to be consistent (within 2%) with three-dimensional measurements. The normal stresses and the Reynolds shear stress for the two- and three-dimensional measurements are compared in Fig. 2b, where the collapse of the datasets across the whole wall-normal range is very good (particularly in the outer region), ensuring that the turbulence statistics in the outer layer are homogenous and, hence, independent of the spanwise direction. As results from two-dimensional PIV data are representative of the bulk flow physics, this justifies performing most of the subsequent analysis (except when clearly stated) on the two-dimensional measurements, which have a higher resolution and are better converged. The difference between the two datasets was found to be of the same order of magnitude as the dispersive stress contribution, although this discrepancy is not purely due to the latter. As is discussed in Placidi (2015), while the dispersive contribution (Raupach and Shaw 1982) is not shown here, it was calculated for each of the statistics following Nikora et al. (2007), and found to be largely negligible in the outer region. Any difference greater than these dispersive contributions (or, equally, than the discrepancies in between the two- and three-dimensional datasets) has to be considered due to the flow physics and it is not due to the spanwise location of measurements.

### Assessing the Flow Regime

**Fig. 3:**
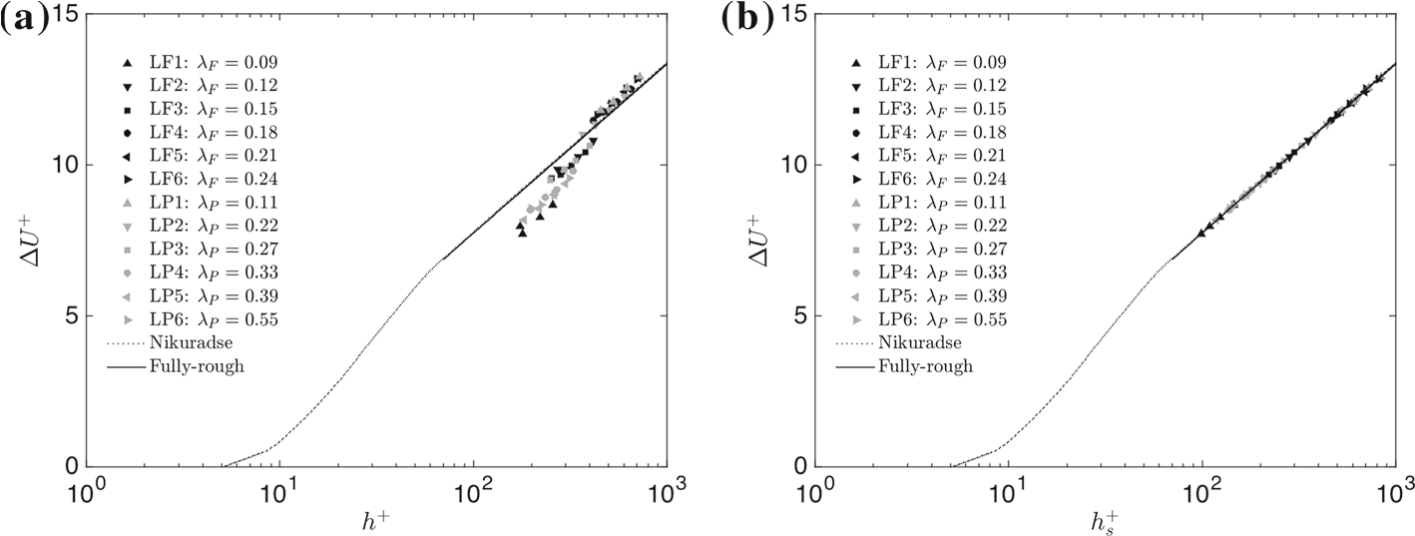
Roughness function $\Delta U^+$ as a function of **a** the roughness Reynolds number $h^+$, and **b** the equivalent sand-grain roughness height $h_s^+$ for all rough surfaces. The dotted line represents a Nikuradse-type roughness for uniform sand, while the solid line is the asymptote for the fully-rough regime

Early experimental studies of rough surfaces were focused on high density rough walls for which a good representation was found to be the equivalent sand-grain roughness height $h_s$ (Nikuradse 1933). As the debate is still open as to which ratio ($\delta /h$ or $\delta /h_s$) is critical when assessing the validity of outer-layer similarity, we estimated both. Nikuradse’s (1933) equivalent sand-grain roughness height can be related to the roughness function $\Delta U^{+}$ following the formulation of Schlichting (1979), and, therefore, to the roughness length $y_{0}$ as shown in Castro (2007), where

2 $$\begin{aligned} \Delta U^{+}=\frac{1}{\kappa }\ln ({h_{s}}^{+})-3.5=B+\frac{1}{\kappa }\ln \left( h^{+}\right) +\frac{1}{\kappa }\ln \left( \frac{y_{0}}{h}\right) , \end{aligned}$$

where *B* is the smooth-wall intercept. It should be noted that the equivalent sand-grain roughness is proportional to the aerodynamic roughness length (with a constant of proportionality that depends on the logarithmic-law constants $\kappa$ and *B*). It was shown in Fig. 5 of Placidi and Ganapathisubramani (2015) that, for all surfaces examined, the measured friction coefficient $C_f$ is constant at different inflow velocities (from 11.5 to 20 m s$^{-1}$) and, therefore, is independent of the Reynolds number, thus satisfying the fully-rough condition. This conclusion is strengthened by examining the roughness function $\Delta U^+$ as a function of the roughness Reynolds number $h^+$, where $\Delta U^+$ is calculated following Eq. 4 in Flack and Schultz (2010) and is reported in Fig. 3a, together with a Nikuradse-type roughness function for uniform sand (Nikuradse 1933). Here, the roughness function for all the examined rough walls appears to be parallel (for $h^{+}>\approx 350$) to the curve for the uniform sand-grain roughness in the fully-rough regime taken from Schultz and Myers (2003). The equivalent sand-grain roughness height $h_s^+$ can instead be calculated using Eq. 5 of Flack and Schultz (2010), with the results shown here in Fig. 3b. All cases clearly collapse onto the asymptote of the fully-rough regime (Flack et al. 2012), which follows a logarithmic-linear relationship between $h_s^+$ and $\Delta U^+$ (Squire et al. 2016).

The sand-grain roughness values are listed in Table 1 1, where it is easy to verify that the ratio $\delta /h$ is roughly constant across all cases, and assumes values in the range $10-12$. As these values are much smaller than 40, the surfaces are to be considered as ‘strong roughnesses’ based on Jimenez’s (2004) definition. This is important as the focus here is exploring the upper limit of outer-layer similarity. Furthermore, when the equivalent sand-grain roughness height is calculated, this also mostly results in surfaces where $\delta /h_s<40$. In this respect, the current data fail to adhere to both Jimenez’s (2004) and Flack’s (2005) characterizations of rough surfaces conforming to outer-layer similarity. However, they are within the limits proposed by Castro (2007) based on cube-roughness data. In a recent study, Squire et al. (2016) also indicate the validity of outer-layer similarity in wall-bounded flows, highlighting that the variance of the streamwise velocity component across rough walls is only expected to conform to Townsend’s similarity hypothesis for flows with $Re_\tau >14000$. However, an outer-layer collapse should also be guaranteed for flows at lower Reynolds number, provided that the fully-rough condition is met. In this respect, given the findings discussed in Fig. 3, and those previously published, profile invariance should be guaranteed for all the cases, which, while failing to adhere to the first condition, are demonstrated to be well within the fully-rough regime.

According to Jackson’s interpretation, it is not uncommon for cases of relatively high roughness ($\delta /h \le 10$) to result in large values of the zero-plane displacement (e.g. for the 10-mm cubes investigated by Castro 2007). This is also the case of this study, as shown in Table 1. Furthermore, if Jackson’s hypothesis that $d/h = 0$ for smooth walls is correct, one would expect that, with increasing roughness density, a situation is reached for which a new shifted smooth-wall is created at $h = d$ (i.e. as $\lambda _\mathrm{F}, \lambda _\mathrm{P}\rightarrow 1$ then $d/h\rightarrow 1$). Therefore, the behaviour found here has to be expected, and is also consistent with most of the morphometric methods (e.g. Macdonald 1998). Having established flow homogeneity and the fact that all surfaces are in the fully-rough regime, the turbulent statistics can be examined in detail.

### Outer-Layer Similarity: Mean and Turbulence Statistics

**Fig. 4:**
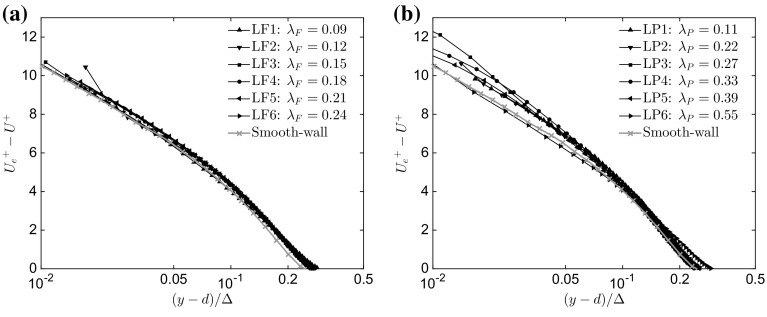
Mean velocity profiles in defect form as a function of **a** $\lambda _\mathrm{F}$ ($\lambda _\mathrm{P}=\mathrm{constant}=0.27$) and **b** $\lambda _\mathrm{P}$ ($\lambda _\mathrm{F}=\mathrm{constant}=0.15$). Only one in every five vectors is plotted for clarity

To assess the validity of outer-layer similarity, one generally starts by examining the mean velocity profiles, which are shown in Fig. 4a in defect form for the LF1 to LF6 cases. To normalize the wall-normal distance, the Clauser scaling parameter is used consistent with Castro (2007) and Amir and Castro (2011), which is defined as $\Delta =(\delta ^{*}U_{e})/U_{\tau }$, where $\delta ^{*}$ is the displacement thickness, and $U_{e}$ is the velocity at the edge of the boundary layer. The mean velocity profiles show a good agreement across all the different cases throughout the entire outer region. The rough-wall cases also appear to collapse fairly well onto the smooth-wall case at a comparable Reynolds number (data from Hutchins et al. 2009). Figure 4b shows the equivalent quantities for the plan solidity cases. Again, a reasonable agreement across all the different cases is shown in the outer region $(y-d)/\Delta \ge 0.1$, however, the quality of the collapse is poorer compared with the results for the frontal solidity cases, and degrades closer to the wall. Furthermore, the rough walls also appear to divert from their smooth counterpart, with a weakening collapse for $(y-d)/\Delta <0.1$, suggesting that outer-layer similarity breaks down with variations in plan solidity, with the smooth wall sitting just below the rough cases. Although not shown here for brevity, the spread in the data across different wall-normal locations and different cases has been calculated and compared with previous experiments reported in the literature (e.g. Amir and Castro 2011), and it was found to be significantly larger than previously reported, indicating a breakdown of outer-layer similarity. Similar comparisons were performed for all quantities reported here, and the assessment of the degree of correspondence with outer-layer similarity is based on these comparisons. Additionally, the dispersive stress contribution was also taken into account; this was generally found to be one order of magnitude smaller than the spread in the data in the outer region, and therefore neglected.

**Fig. 5:**
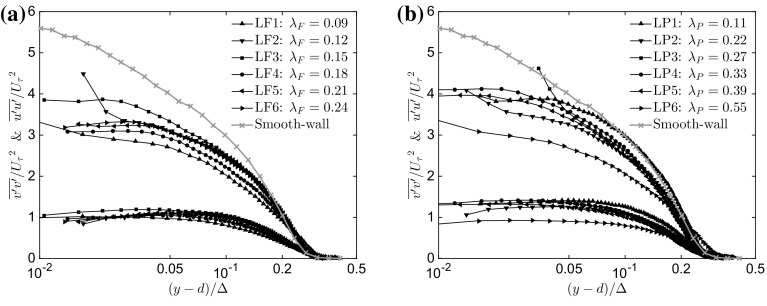
Wall-normal variation of the streamwise component of turbulence intensity ($\overline{u'u'}/{U_{\tau }}^2$) and wall-normal turbulence intensity ($\overline{v'v'}/{U_{\tau }}^2$) as a function of **a** $\lambda _\mathrm{F}$ ($\lambda _\mathrm{P}=\mathrm{constant}=0.27$) and **b** $\lambda _\mathrm{P}$ ($\lambda _\mathrm{F}=\mathrm{constant}=0.15$). Only one in every five vectors is plotted for clarity

Figure 5a shows the streamwise and the wall-normal fluctuating velocity components for different values of $\lambda _\mathrm{F}$. The profiles of the rough-wall streamwise component of turbulent intensity collapse reasonably well for $(y-d)/\Delta >0.2$, whilst major differences appear closer to the wall. The LF1 and LF3 cases exhibit the largest differences and departure from the other cases for $(y-d)/\Delta <0.2$. However, the profiles of the streamwise fluctuating velocity component for the rough walls are significantly lower in value than those of the smooth case for $(y-d)/\Delta <0.15$, which is partially due to the much higher friction velocity generated by the rough walls.^2^ The wall-normal component of turbulence intensity shows a better collapse (in % spread) throughout the entire range of wall-normal locations in comparison with the previously reported percentage spread in Amir and Castro (2011), although smooth-wall data for this velocity component are not available. Figure 5b shows the streamwise and the wall-normal fluctuating velocity components for different values of $\lambda _\mathrm{P}$. Both profiles of the streamwise and wall-normal components of the turbulence intensity show a more evident lack of similarity across the entire wall-normal range, which confirms the lack of similarity found in the previous studies, especially for two-dimensional roughness elements (Volino et al. 2009) and for low values of frontal solidities (Ganapathisubramani and Schultz 2011). The velocity fluctuations for the smooth-wall case sit just above both the rough-wall cases in accordance with Keirsbulck et al. (2002).

**Fig. 6:**
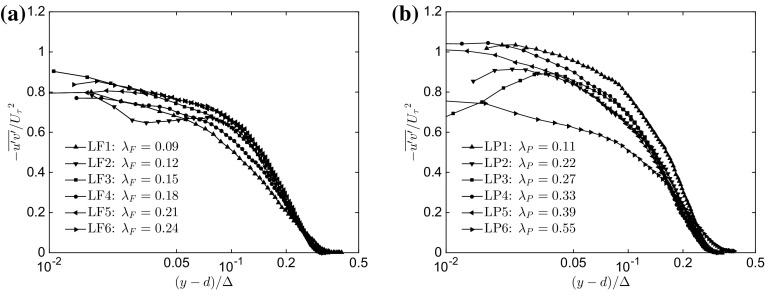
Wall-normal variation of Reynolds shear stress ($-\overline{u'v'}/{U_{\tau }}^2$) as a function of **a** $\lambda _\mathrm{F}$ ($\lambda _\mathrm{P}=\mathrm{constant}=0.27$) and **b** $\lambda _\mathrm{P}$ ($\lambda _\mathrm{F}=\mathrm{constant}=0.15$). Only one in every five vectors is plotted for clarity

To further analyze the collapse of the different rough-wall profiles, Fig. 6a, b present the Reynolds shear stress for the different $\lambda _\mathrm{F}$ and $\lambda _\mathrm{P}$ cases, respectively. The shear stress seems to be the most affected quantity by the roughness, where a lack of collapse throughout the entire $(y-d)/\Delta$ range is shown for both $\lambda _\mathrm{F}$ and $\lambda _\mathrm{P}$. Particularly significant is the difference for the LP6 case, which shows much lower turbulence stresses across almost the entire wall-normal range. It is perhaps worth discussing why the normalized Reynolds shear-stress profiles displayed in Fig. 6 do not reach a plateau at unity, as is commonly reported in the literature. Cheng and Castro (2002) showed that for boundary-layer flows over staggered arrays of cubical elements, extrapolating the skin friction from Reynolds shear-stress profiles underestimates it by $\approx 25\%$, for which they recommended applying the numerical correction contained in Reynolds and Castro (2008). As our study has been conducted in the same facility and has very similar surface morphologies ($h = 10$ mm) as those in Cheng and Castro (2002), we believe that a similar correction should be applied. This is confirmed by the fact that only after this correction is applied do the results of the total stress method match the drag-balance results (within $5\%$), which raises new questions about the validity of outer-layer similarity in previous studies that have used indirect methods to obtain the skin friction on large roughness elements. Here, we have used a drag balance for the estimation of $U_\tau$ to overcome this shortcoming.

### Outer-Layer Similarity: Diagnostic Plot

**Fig. 7:**
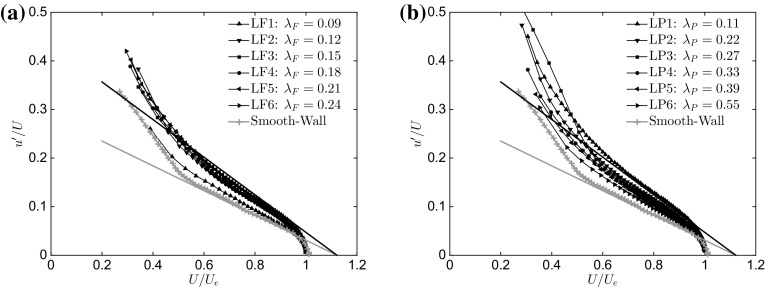
Diagnostic plot for the different rough surfaces as a function of **a** $\lambda _\mathrm{F}$ ($\lambda _\mathrm{P}=\mathrm{constant}=0.27$) and **b** $\lambda _\mathrm{P}$ ($\lambda _\mathrm{F}=\mathrm{constant}=0.15$). The black solid line represents the fully-rough regime from Castro et al. (2013), while the grey line is the smooth-wall limit reported by Alfredsson et al. (2011). Only one in every five vectors is plotted for clarity

Thus far, the topic of outer-layer similarity has been discussed according to the classical definition, i.e. the variation of mean and fluctuating quantities with the appropriately-scaled wall-normal distance. Recently, an alternative solution using the ‘diagnostic plot’ has been proposed (Alfredsson and Örlü 2010; Alfredsson et al. 2011; Castro et al. 2013) to find that fully-rough data scaled in diagnostic form appear to collapse in the outer layer, which eliminates the uncertainty in determining $U_\tau$, the actual wall-normal position and the zero-plane displacement. Figure 7a, b presents the current data for $\lambda _\mathrm{F}$ and $\lambda _\mathrm{P}$ cases, respectively, in the diagnostic form, where data in the fully-rough regime should collapse onto the solid black line, which is a linear best fit from various datasets in the fully-rough regime taken from Castro et al. (2013). The figures also show the best fit in the outer layer for smooth walls (grey line), which is in good agreement with the smooth-wall data of Hutchins et al. (2009). However, the collapse across the current cases is poor, with the lack of collapse more pronounced in the $\lambda _\mathrm{P}$ cases (Fig. 7b), which is consistent with our findings based on classical scaling, and is further proof of the breakdown of outer-layer similarity in these flows. Note that our data do not collapse on the trend line for the fully-rough regime (black solid line), but fall predominantly between the smooth and the fully-rough asymptotes; this, Castro et al. (2013) have argued, is a common behaviour for transitionally rough flows. However, the value for $y^+_0$ in our dataset is significant ($y^+_0>10$), and comparable to their cases that follow the fully-rough trend lines. Moreover, the spatial resolution of our measurements is comparable to previous measurements that show outer-layer similarity. Therefore, for these reasons, the lack of collapse displayed by our data must be interpreted as a breakdown of outer-layer similarity, which raises the possibility that the fully-rough asymptote in the diagnostic plot is, in fact, dependent on other additional parameters, such as surface-roughness morphology, $\delta /h$, $h^+$, $\delta /y_0$, which is a possibility recognized in Castro et al. (2013). The most significant difference between our data and those of Castro et al. (2013) is that $\delta /h$ is consistently around 10 (compared with the much higher values for various datasets in their paper). This suggests that the diagnostic plot (and thus its asymptotes) is more sensitive to surface parameters for low values of $\delta /h$. Our analysis seems to suggest that the breakdown of outer-layer similarity depends not only on the relative height of the roughness elements (i.e. $\delta /h$ or $\delta /h_s$), but also strongly on the local characteristics of the wall. Therefore, applying Townsend’s hypothesis to any wall below a certain height of the roughness elements, irrespective of its characteristics, may lead to erroneous conclusions, which is consistent with the recent suggestions of Squire et al. (2016).

Before discussing what parameter governs this lack of outer-layer similarity, it is important to consider the nature of the roughness in terms of its effect on the nearby flow. In the limit of very dense and shallow roughness ($h<<\delta$), as in the case of closely packed sand grains, a good agreement with outer-layer similarity has generally been reported. However, in the limit of very sparse roughness (e.g. when both $\lambda _\mathrm{F}$ and $\lambda _\mathrm{P}$ $\rightarrow 0$), the flow resembles a situation more similar to that of flow over isolated obstacles (or bluff bodies), and the universality of the flow is bound to break down, as this is locally perturbed. Here, we consider the validity of outer-layer similarity for flows over severe roughness for sparse to dense regimes, which is chosen to explore the upper bound of the validity of outer-layer similarity, and to compare with the previous studies on sparse cubes discussed in Sect. 1. Despite these conditions, it is important to recall that, for all surfaces here, the extent of the roughness sublayer was calculated in Placidi and Ganapathisubramani (2015), with the flow found to be homogeneous across the spanwise direction (when outside the roughness sublayer). Although in the sparse regime, the cases examined are far from being flows over isolated obstacles.

Finally, considering the shape of the roughness used here, LEGO™ bricks are characterized by pins on the top surface much smaller than the blocks, and with a characteristic length scale one order of magnitude smaller. A similar roughness was investigated by Colebrook and White (1937) who found that a small background roughness superimposed on sparse large-scale morphology can significantly modify the skin friction. However, Schultz and Flack (2005) repeated a similar experiment concluding that ‘the addition of a secondary roughness length scale had no effect on the Reynolds stresses throughout the entire boundary layer, indicating that the larger-scale roughness had a dominant effect on the turbulence’. They also noted that Colebrook and White (1937) did not have sufficient data in the fully-rough regime, which may have led the small-scale roughness having an effect on the mean flow. The surfaces examined here do not suffer from this limitation as they are in fully-rough conditions. Therefore, the breakdown of outer-layer similarity should be attributed to the large-scale roughness (i.e. the bricks) and not to the presence of the finer-scale features.

### Reconciling the Lack of Outer-Layer Similarity

**Fig. 8:**
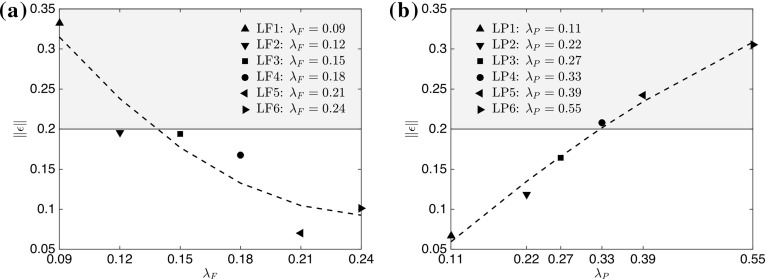
The norm $\Vert \epsilon \Vert$ of the deviation from the turbulent asymptote in Fig. 7 as a function of **a** $\lambda _\mathrm{F}$ ($\lambda _\mathrm{P}=\mathrm{constant}=0.27$) and **b** $\lambda _\mathrm{P}$ ($\lambda _\mathrm{F}=\mathrm{constant}=0.15$) calculated in the range $0.6<U/Ue<0.9$. Black dashed lines indicate a second-order polynomial fit of the data points. The grey shaded area represents data that do not satisfy the condition $\Vert \epsilon \Vert \le 20\%$ as discussed in Sect. 3.5

To further investigate parameters that are responsible for the breakdown of outer-layer similarity, the data in Fig. 7 can be conditioned in terms of the deviation from the suggested rough-wall asymptote in a procedure similar to that described in Hanson and Ganapathisubramani (2016). The deviation is quantified by the means of the norm $\Vert \epsilon \Vert$ in between the measured data points and the fully-rough asymptote within the range $0.6<U/Ue<0.9$, which is purely based on the previous collapse of the data presented in Castro et al. (2013), with the results shown in Fig. 8. It is clear how the data show trends for both frontal and plan solidity cases. Increasing the frontal solidity (from case LF1 to case LF6) results in a smaller norm, i.e. a smaller deviation from the empirically defined asymptote, with the same effect obtained instead for a decrease in plan solidity (going from case LP6 to LP1). This means that, by adding roughness in one case (by increasing $\lambda _\mathrm{F}$), we introduce turbulent fluctuations with a self-similar character, while in the other case (by increasing $\lambda _\mathrm{P}$), the associated turbulent fluctuations are highly dependent on the details of the local morphology, contributing to the breakdown of outer-layer similarity.

As it is clear from the above discussion that the deviation from outer-layer similarity could be related to the surface morphology, it is useful to examine the possibility of correlating the lack of similarity with a specific surface-roughness parameter. Of the different morphometric parameters of the surface examined, the shelter solidity $\lambda _\mathrm{S}$ was found to be the most useful in distinguishing the surfaces based on their deviation from outer-layer similarity, which is defined as

3 $$\begin{aligned} \lambda _\mathrm{S} = \frac{A_\mathrm{T} - A_\mathrm{S}}{A_\mathrm{T}}, \end{aligned}$$

where $A_\mathrm{T}$ is the total plan area of a repeating unit and $A_\mathrm{S}$ is the ‘sheltered’ plan area of the roughness in a repeating unit. This sheltered area is identified by the red, shaded region in Fig. 1, while the total plan area is simply the whole area comprising a repeated unit. The sheltered plan area $A_\mathrm{S}$ can be interpreted as the area sheltered by the roughness elements within the repeated unit, and is very similar to the ‘effective shelter plan area’ previously outlined in the literature. In Raupach (1992), the definition of the shelter area is based on the wake growth of an individual roughness element. Here, we do not assume wake growth/decay, but simply approximate the sheltered regions with straight lines (in the direction of the flow) within the repeating unit. Consequently, $A_\mathrm{S}$ can also be interpreted as the wetted plan area downstream of the roughness elements within a repeated unit.

**Fig. 9:**
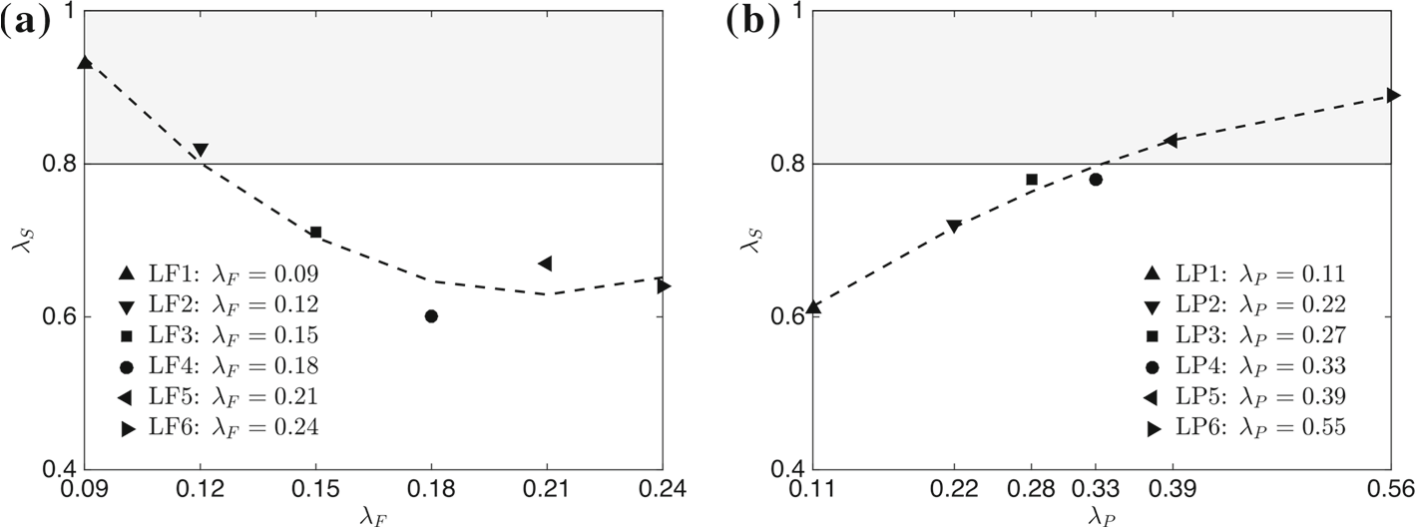
Shelter solidity $\lambda _\mathrm{S}$ as a function of **a** $\lambda _\mathrm{F}$ ($\lambda _\mathrm{P}=\mathrm{constant}=0.27$) and **b** $\lambda _\mathrm{P}$ ($\lambda _\mathrm{F}=\mathrm{constant}=0.15$). Black dashed lines indicate a second-order polynomial fit of the data points. Grey shaded area represents data that do not satisfy the condition $\lambda _\mathrm{S}\le 80\%$

Furthermore, another distinction with the previous definition is that, here, we do not include the area occupied by the elements themselves when calculating the sheltered area, which helps to eliminate some limitations of the previous definition found in Raupach (1992). Figure 9 shows the variation of $\lambda _\mathrm{S}$ for different $\lambda _\mathrm{F}$ and $\lambda _\mathrm{P}$ cases. It is striking how the trends found for the deviation from the similarity asymptotes (in Fig. 8) are captured by this parameter. For all the cases reported here, it is found that for $\lambda _\mathrm{S} \ge 0.8$, the statistics in Fig. 7 show a high degree of deviation from outer-layer similarity. This condition is indicated by the grey, shaded area in both figures. Therefore, any surface within this shaded region is expected to show a significant deviation from the rough-wall asymptote suggested by Castro et al. (2013) and, generally, result in the breakdown of outer-layer similarity. The condition $\lambda _\mathrm{S} \ge 0.8$, which corresponds to $\Vert \epsilon \Vert \ge 20\%$ in Fig. 8, represents situations where the total sheltered area due to the rough elements is below one-fifth of the total plan area of the repeated unit.

The above correlation does not explain the breakdown of outer-layer similarity observed in two-dimensional spanwise roughness, such as bars/groves (Keirsbulck et al. 2002; Volino et al. 2009, 2011). The value of $\lambda _\mathrm{S}$ for two-dimensional roughness depends on the pitch-to-width (*p* / *k*) ratio of the roughness. The plan area is proportional to the pitch, while the sheltered area (as per our definition) is given by the difference between the pitch and width ($p-k$). For $p > k$, $\lambda _\mathrm{S}$ approaches zero with increasing *p* / *k*. However, if the roughness is in the form of elongated roughness elements (i.e. $p/k<< 1$), then the values of $\lambda _\mathrm{S}$ are closer to unity again, which suggests that the lack of outer-layer similarity is likely to occur for both large and small values of $\lambda _\mathrm{S}$.

**Fig. 10:**
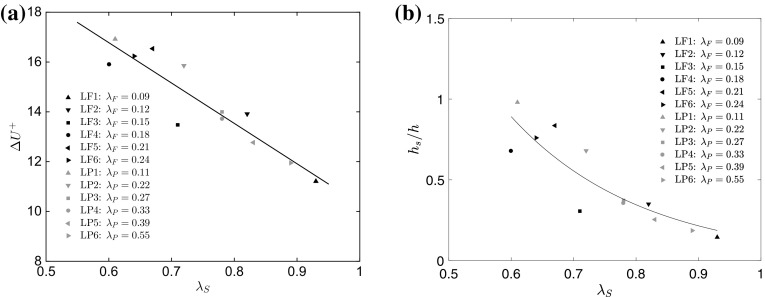
Dependence of the **a** roughness function $\Delta U^+$, and **b** the quantity $h_s/h$ on the shelter solidity for all cases. Black lines indicate a first-order polynomial and exponential fits of the data, respectively

Finally, our proposed surface parameter can also be used to reconcile the variations in surface drag. Figure 10a shows the dependence of the roughness function on $\lambda _\mathrm{S}$, where a linear relationship (inverse proportionality) is found to be representative of most of the data. We also plot the quantity $h_s/h$ as a function of $\lambda _\mathrm{S}$ in Fig. 10b, where the shelter solidity seems to adequately capture the behaviour of the normalized equivalent roughness height, which is very similar to the relationship between $h_s/h$ and the effective slope found in previous studies on irregular roughness (Napoli et al. 2008;Yuan and Piomelli 2014). Further studies must address the robustness of this new parameter, and potentially calibrate it to different geometries and less severe roughness.

### Similarity of Proper Orthogonal Decomposition Modes

**Fig. 11:**
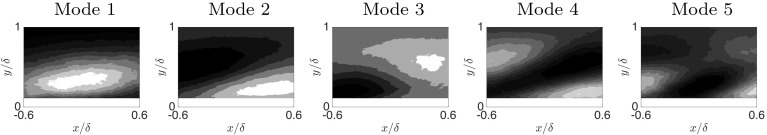
The first five low-order POD modes for the case LF5 calculated for the combined $(u',v')$ field in the (*x*, *y*) plane. The flow is from left to right

A snapshot-based proper orthogonal decomposition (POD) analysis on the basis of Berkooz et al. (1993) was presented in Placidi and Ganapathisubramani (2015). As the energy contained in the POD modes depends on the local spatial resolution of the measurements (see Appendix), the current data have been filtered to match the local resolution at $l_{2D}^{+}=45$. The field-of-view of combined $(u',v')$ data across the different cases is also matched to the region $-0.6\delta<x<0.6\delta$ and $1.5h\le y\le \delta$ (streamwise $\times$ wall-normal) to allow for meaningful comparisons. Figure 11 shows the first five most energetic POD modes. While most of these modes were discussed in Placidi and Ganapathisubramani (2015), they are also presented here to introduce the subsequent analysis. Placidi and Ganapathisubramani (2015) also showed that all frontal and plan solidity cases present similar POD mode shapes across all sparse to dense regimes, which is remarkable considering how different the wall topologies are across the cases examined. The more quantitative POD information reported in Placidi (2015) is omitted here for brevity.

**Fig. 12:**
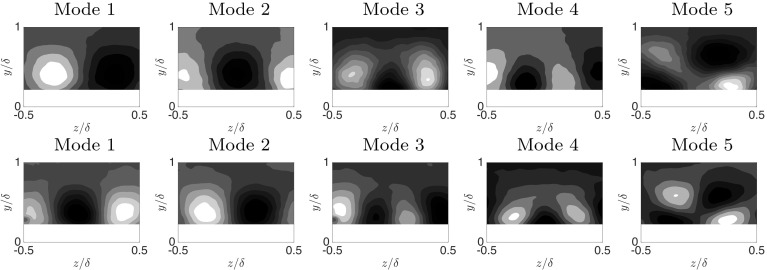
The first five low-order POD modes for both the frontal and plan solidities calculated on the combined $(u',v',w')$ field in the (*y*, *z*) plane for the cases LF5 and LP5 shown as the top and bottom rows, respectively. The flow is from left to right

To further comment on the similarity of POD modes, the same technique has been applied to the cross-plane PIV, for which the full velocity vector $(u',v',w')$ is available, with similar boundaries to the previous case used to compute the POD modes. The results are shown in Fig. 12 for both $\lambda _\mathrm{F}$ and $\lambda _\mathrm{P}$, which are presented on the top and bottom rows, respectively. Compared with results on the (*x*, *y*) plane (see Fig. 11), these cross-plane mode shapes appear to be generally smaller, which is not surprising given the added contribution of the $(w')$ field, which is bound to leave an imprint on the structures of the POD modes. Despite some changes in the hierarchy of the modes (i.e. mode 1 and 2 and mode 3 and 4 between frontal and plan solidity cases), an exceptional degree of similarity is still evident across the whole dataset. The remaining cases (omitted here) look indistinguishable (in both size and shape) from those presented. The mode swap could be due to the different spanwise arrangement of the elements and the higher sensitivity of the (*y*, *z*) data to the heterogeneity within the roughness sublayer, as further discussed in Placidi and Ganapathisubramani (2015). Alternatively, a phase difference between the combination of the first and second modes across experiments may also explain the discrepancy. Nevertheless, changes in frontal and plan solidities do not seem to affect the shape of the energetic POD modes, which are found to be universal across the different wall morphologies. This is a testimony to the spatial universality of the turbulence structure over rough walls. In this respect, the POD mode criteria can be considered as a spatial variant of ‘spectral’ similarity.

### Reduced-Model Similarity

To address those particularly sized structures responsible for the lack of outer-layer similarity, POD modes can be used to bandpass filter the velocity fields to obtain a spatially-filtered representation of the original field (Wu and Christensen 2010; Wu 2014). Bandpass-filtered velocities are generated after reconstructing each individual fluctuating velocity field by projecting it onto the most energetic $\phi _{i-th}$ modes containing $50\%$ of the total turbulent kinetic energy (Wu and Christensen 2010), which is denoted 0.5*E*. While the number of modes required to reach this arbitrary limit of energy varies for the different cases, the comparison is reasonable as it is based on the definition of POD basis.

**Fig. 13:**
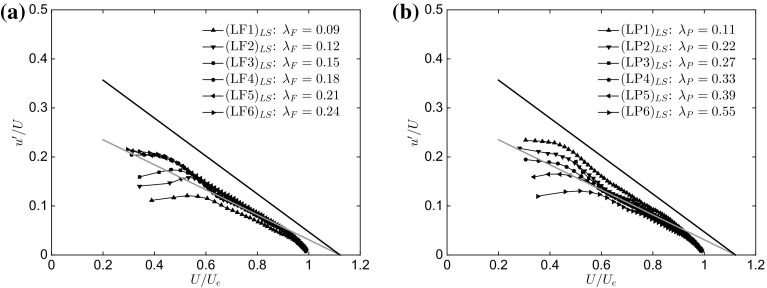
Diagnostic plot for the large-scale fields reconstructed from a reduced model (i.e. 0.5*E*) of the different rough surfaces as a function of **a** $\lambda _\mathrm{F}$ ($\lambda _\mathrm{P}=\mathrm{constant}=0.27$) and **b** $\lambda _\mathrm{P}$ ($\lambda _\mathrm{F}=\mathrm{constant}=0.15$). The black solid line represents the fully-rough regime from Castro et al. (2013), while the grey line is the smooth-wall limit reported by Alfredsson et al. (2011). Only one in every five vectors is plotted for clarity

Once the reduced model is constructed, the turbulence statistics for the large-scale contributions are examined, which allows determination of the breakdown of the outer-layer similarity due to the relative contributions of the large and small scales. The POD calculation is applied to a region of approximately $\delta \times \delta$ (streamwise $\times$ wall-normal) field-of-view on the $(u')$ field. Results of this procedure for the streamwise component of the fluctuations are presented in Fig. 13, with **a** showing results for the variation of $\lambda _\mathrm{F}$, while **b** concerns the $\lambda _\mathrm{P}$ cases. This ‘diagnostic plot’ form is chosen to represent this information since it eliminates the need to define an ad hoc scaling for the velocities (see discussion in Sect. 3.3), especially an appropriate skin friction for the reduced-order models, which is not obvious and needs to be defined. In all cases, the reduced model statistics (i.e. subscript $_{LS}$) seem to show better self-similar properties when compared with the unfiltered data in Fig. 7. It is also interesting to note how all cases have moved towards the smooth-wall asymptote (grey line) and collapsed onto it for $0.6<U/U_{e}<0.85$. This suggests that the small-scale turbulence generated by the roughness (which has been filtered out) is highly sensitive to the surface morphology. Previous studies (Hong et al. 2011) have also discussed how the small-scale turbulence can contain a clear roughness signature across the entire wall-normal range, despite the fact that the imprint of the roughness is reduced in the outer layer, in accordance with outer-layer similarity. However, this is in contrast with Wu and Christensen (2010) who found that the small scales should be insensitive to roughness effects. Further studies are required to clarify this point.

### Two-Point Velocity Correlations Similarity

**Fig. 14:**
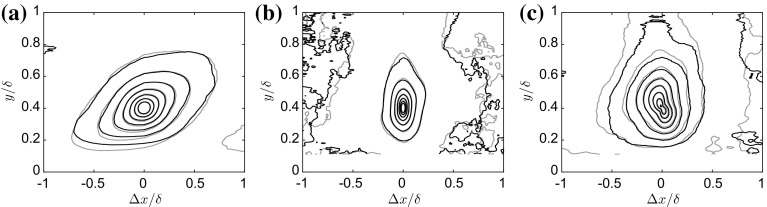
Two-point correlations centred at $y/\delta =0.4$. $\mathbf{a}~ R_{u'u'}, \mathbf{b}~ R_{v'v'}$ and $\mathbf{c}~R_{u'v'}$. The innermost contours for $R_{u'u'}$ & $R_{v'v'}$ is 0.8 and contour spacing is 0.1. For $R_{u'v'}$, the innermost contour is − 0.4 and the spacing is 0.05. Grey is used for the LF3 case, whist black is used for the LP3 case. The flow is from left to right

To further investigate the universality of the large-scale turbulence across rough walls, two-point velocity correlations at a chosen wall-normal location ($y=0.4\delta$) are evaluated following Ganapathisubramani et al. (2005). Figure 14a shows contours of the two-point correlations of the streamwise component of fluctuating velocities. The two cases LF3 and LP3 are plotted in grey and black, respectively, and superimposed to facilitate comparison. A large forward-leaning structure of positive correlation is visible in both cases, which is consistent with recurrent features in the instantaneous snapshots (not shown), as well as with several previous studies (Ganapathisubramani et al. 2003, 2005; Wu and Christensen 2006; Volino et al. 2007, 2009; Dennis and Nickels 2011). Here, the two cases are found to be virtually indistinguishable within experimental uncertainty. The angle of inclination of these structures was found to be approximately $15^\circ \pm 3^\circ$ for all cases, which is in line with previous observations.

The two-point correlation of the wall-normal fluctuations $R_{v'v'}$ is presented in Fig. 14b, where the correlation structure is much more compact in the streamwise direction as previously shown (Volino et al. 2009). This is not surprising given that the streamwise velocity component depends on the advective speed of the hairpin packets, whereas the wall-normal velocity component does not (Volino et al. 2007). Once again, the two cases show remarkable collapse. Finally, the two-point correlation of the Reynolds shear stress $R_{u'v'}$ is presented in Fig. 14c, where, in agreement with that reported in the literature, a backwards-leaning structure of strong negative correlation is found whose extent is larger than the wall-normal correlation, but smaller than the streamwise coherence. The two cases still show very good agreement despite the fact that the experimental uncertainty in this quantity is larger.

We stress that, although the correlation maps are shown for just two cases, the findings are consistent across all other cases, with the shapes consistent across the different roughness morphologies, which further supports the presence of self-similarity in the spatial structure of turbulence, despite dissimilar statistics. This is perhaps understandable due to the fact that the large scales of the turbulence are the major contributors to the correlation structure and lower-order POD modes, while the turbulence statistics are locally influenced by the small scales, and thus intimately linked to the local wall topology.

## Conclusions

Experiments were carried out on fully-rough boundary layers developed over large roughness ($\delta /h \approx$ 10), with twelve different surface conditions explored using LEGO™ bricks of uniform height. Six cases presented a fixed-plan solidity while varying the frontal density, and a further six obtained vice versa. These contribute to a unique and extensive dataset exploring the impact of surface morphology on flow statistics and their universality.

Mean velocity profiles in defect form conform to outer-layer similarity for different $\lambda _\mathrm{F}$ cases, while the collapse degrades marginally across $\lambda _\mathrm{P}$ cases, which suggests that, for a given frontal blockage, the plan arrangement of the roughness leads to the violation of outer-layer similarity. The streamwise and wall-normal turbulent intensities, as well as Reynolds shear stresses, show a lack of outer-layer similarity for almost all cases considered. So for these rough surfaces with a significant relative height ($\delta /h\approx 10$), the flow does not follow outer-layer similarity, particularly in higher-order quantities. This disagrees with Amir and Castro (2011) who suggested that outer-layer similarity is present down to $\delta /h\approx$ 7 in cubic roughness. Therefore, the maximum relative height of the roughness elements for which outer-layer similarity hold depends not only on the ratios $\delta /h$ or $\delta /h_s$, as previously suggested (Jimenez 2004; Flack et al. 2005), but also on the local roughness morphology, as well as the geometry of the individual roughness elements. A new geometric parameter named the shelter solidity $\lambda _\mathrm{S}$ is proposed to correlate the lack of similarity to features of the surface morphology. It was found that, if the sheltered area within one repeated roughness unit is less than some $20\%$ of its total plan area (so that $\lambda _\mathrm{S}>0.8$), then the flow over that surface does not conform to outer-layer similarity.

Proper orthogonal decomposition (POD) analysis shows that the significantly different rough walls exhibit virtually identical POD modes both in the (*x*, *y*) and (*y*, *z*) planes, suggesting that, although the spatial organisation of the turbulence appears to be universal across all rough-wall boundary layers (in accordance with the previous literature), its strength is modulated by the wall-local morphology. Therefore, the similarity of POD mode shapes can be considered as a relaxed form of Townsend’s hypothesis immune from problems arising from the inappropriate scaling of the velocity profiles. The reduced-order model statistics derived from the POD analysis seem to suggest that the turbulence at small scales, which can be linked to the local surface morphology, play a substantial role in the breakdown of outer-layer similarity.

Finally, it is confirmed that the turbulence at large scales truly behaves in a universal manner across rough walls, as two-point correlations of velocity fluctuations are shown to be virtually indistinguishable across frontal and plan solidity cases, which corroborates this universality.

## Figures and Tables

**Fig. 15:**
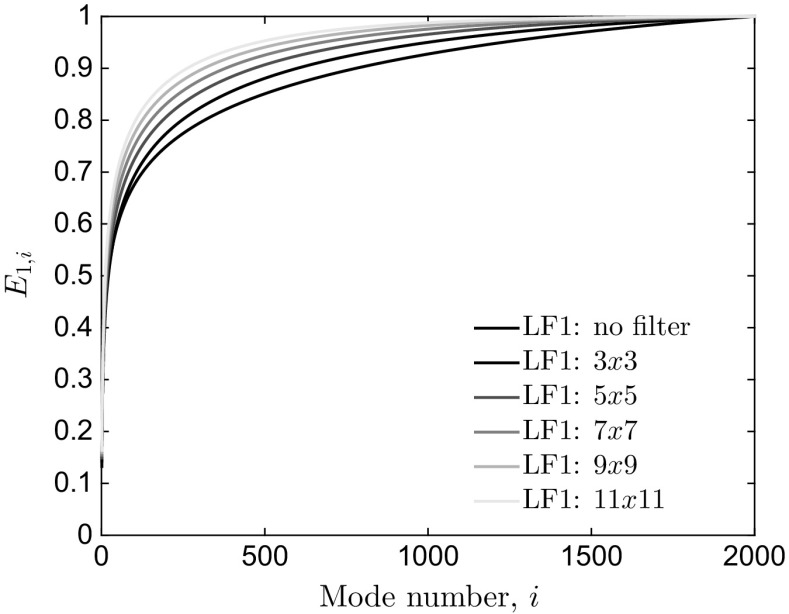
Effect of filter size on the cumulative energy content $E_{1,i}$ versus mode number for the LF1 case

## Appendix: Effect of Spatial Resolution on the Proper Orthogonal Decomposition Analysis

To demonstrate that the turbulent statistics, and, in particular, the results obtained via the POD analysis depend on the local spatial resolution of the dataset, one case (LF1) has been digitally filtered with increasingly bigger kernel size, and the POD analysis has been performed on the filtered dataset. Results from filtered and unfiltered data are then compared. Kernel sizes from 3$\times$3 to 11$\times$11 are used for this purpose, with the results of this procedure shown in Fig. 15. The cumulative energy *E* as a function of the mode number is clearly shown to be dependent on the resolution (i.e. on the kernel size). A larger amount of energy is locked into each lower-order mode with increasing filter size. The fractional energy contribution $E_{i}$ of the *i*th POD mode $\phi _{i}$ to the total *E* is also affected by the dataset resolution as shown in Table 2. The energy in the first mode goes from $E_1=12\%$ for the unfiltered case to $E_1=16\%$ when an 11$\times$11 filter is applied. Additionally, the cumulative contribution of the first five modes $E_{1,5}$ also varies from $32\%$ to $37\%$. It is, therefore, obvious that spatial filtering the data (hence reducing its resolution) redistributes the relative energy toward the lower-order modes (and hence the large scales). This process occurs because the small-scale flow features are removed by the filtering procedure, and hence, the first few modes represent proportionately more and more of the total energy of the flow (Pearson et al. 2013). Therefore, when comparing statistics of the POD results across different datasets, it is important to match the resolution to make appropriate comparisons.

**Table 2:** Effect of filter size on fractional turbulent kinetic energy and cumulative turbulent kinetic energy contents

	Dataset	Filter	$l^+$	$E_i$ ($i=1, 2, 3, 4, 5$)	$E_{1,i}$ ($i=5, 100, 500, 1000$)
	LF1	No filter	28	12–7–6–4–3	32–66–84–92
	LF1	$3\times 3$	84	14–7–5–4–3	32–69–88–95
	LF1	$5\times 5$	140	15–7–5–4–3	33–72–91–97
	LF1	$7\times 7$	196	15–7–5–4–3	35–75–92–97
	LF1	$9\times 9$	252	16–7–5–4–3	36–77–94–98
	LF1	$11\times 11$	308	16–8–6–4–3	37–79–95–99

## Footnotes

1 Corrigendum: we are grateful to the anonymous reviewer who pointed out an error in the calculation of the plan solidity value for the LP6 case in Placidi and Ganapathisubramani (2015). The value previously reported $\lambda _\mathrm{P}=0.44$ should be replaced by $\lambda _\mathrm{P}=0.55$. All the conclusions of our previous paper remain unchanged.

2 It is important to comment on the apparent lack of the inner peak in the streamwise fluctuating velocity component for the smooth wall. This is purely due to an artefact of the scaling of the ordinate axis in Fig. 5, where the rough-wall data are only available for $y\ge h$. The inner peak in smooth walls is commonly found at a much lower wall-normal range (i.e. $y^+\approx 15$), as in Hutchins et al. (2009).

## References

[CR1] Acharya M, Bornstein J, Escudier MP (1986). Turbulent boundary layers on rough surfaces. Exp Fluids.

[CR2] Adrian RJ, Westerweel J (2011). Particle image velocimetry.

[CR3] Alfredsson HP, Örlü R (2010). The diagnostic plot—a litmus test for wall bounded turbulence data. Eur J Mech B/Fluids.

[CR4] Alfredsson HP, Segalini A, Örlü R (2011). A new scaling for the streamwise turbulence intensity in wall-bounded turbulent flows and what it tells us about the “outer” peak. Phys Fluids.

[CR5] Amir M, Castro IP (2011). Turbulence in rough-wall boundary layers: universality issues. Exp Fluids.

[CR6] Andreopoulos J, Bradshaw P (1981). Measurements of turbulence structure in the boundary layer on a rough surface. Boundary-Layer Meteorol.

[CR7] Antonia RA, Luxton RE (1971). The response of a turbulent boundary layer to a step change in surface roughness Part 1. Smooth to rough. J Fluid Mech.

[CR8] Bakken OM, Krogstad PA, Ashrafian A, Andersson HI (2005). Reynolds number effects in the outer layer of the turbulent flow in a channel with rough walls. Phys Fluids.

[CR9] Benedict LH, Gould RD (1996). Towards better uncertainty estimates for turbulence statistics. Exp Fluids.

[CR10] Benson J (2005) Boundary Layer response to a change in surface roughness. Ph.D. thesis, University of Reading, Department of Meteorology, The University of Reading

[CR11] Berkooz G, Holmes P, Lumley JL (1993). The proper orthogonal decomposition in the analysis of turbulent flows. Annu Rev Fluid Mech.

[CR12] Bhaganagar K, Kim J, Coleman G (2004). Effect of roughness on wall-bounded turbulence. Flow Turbul Combust.

[CR13] Castro IP (2007). Rough-wall boundary layers: mean flow universality. J Fluid Mech.

[CR14] Castro IP, Segalini A, Alfredsson HP (2013). Outer-layer turbulence intensities in smooth- and rough-wall boundary layers. J Fluid Mech.

[CR15] Cheng H, Castro IP (2002). Near wall flow over urban-like roughness. Boundary-Layer Meteorol.

[CR16] Cheng H, Hayden P, Robins AG, Castro IP (2007). Flow over cube arrays of different packing densities. J Wind Eng Ind Aerodyn.

[CR17] Coceal O, Belcher SE (2004). A canopy model of mean winds through urban areas. Q J R Meteorol Soc.

[CR18] Colebrook CF, White CM (1937). Experiments with fluid friction in roughened pipes. Proc R Soc.

[CR19] Dennis DJC, Nickels TB (2011). Experimental measurement of large-scale three-dimensional structures in a turbulent boundary layer. Part 1. Vortex packets. J Fluid Mech.

[CR20] Efros V (2011) Scructure of turbulent boundary layer over a 2-D roughness. Ph.D. thesis, Norwegian University of Science and Technology

[CR21] Flack KA, Schultz MP (2010). Review of hydraulic roughness scales in the fully rough regime. J Fluid Eng.

[CR22] Flack KA, Schultz MP, Shapiro TA (2005). Experimental support for Townsend’s reynolds number similarity hypothesis on rough walls. Phys Fluids.

[CR23] Flack KA, Schultz MP, Rose WB (2012). The onset of roughness effects in the transitionally rough regime. Int J Heat Fluid Flow.

[CR24] Ganapathisubramani B, Schultz MP (2011) Turbulent boundary layer structure over sparsely distributed roughness. In: 8th International symposium on turbulence and shear flow phenomena, Poitiers, France August 28–30

[CR25] Ganapathisubramani B, Longmire EK, Marusic I (2003). Characteristics of vortex packets in turbulent boundary layers. J Fluid Mech.

[CR26] Ganapathisubramani B, Hutchins N, Hambleton WT, Longmire EK, Marusic I (2005). Investigation of large-scale coherence in a turbulent boundary layer using two-point correlations. J Fluid Mech.

[CR27] Grimmond CSB, Oke TR (1999). Aerodynamic properties of urban areas derived, from analysis of surface form. J Appl Meteorol.

[CR28] Hagishima A, Tanimoto J, Nagayama K, Meno S (2009). Aerodynamic parameters of regular arrays of rectangular blocks with various geometries. Boundary-Layer Meteorol.

[CR29] Hanson RE, Ganapathisubramani B (2016). Development of turbulent boundary layers past a step change in wall roughness. J Fluid Mech.

[CR30] Hong J, Katz J, Schultz MP (2011). Near-wall turbulence statistics and flow structures over three-dimensional roughness in a turbulent channel flow. J Fluid Mech.

[CR31] Hong J, Katz J, Meneveau C, Schultz MP (2012). Coherent structures and associated subgrid-scale energy transfer in a rough-wall turbulent channel flow. J Fluid Mech.

[CR32] Hutchins N, Nickels TB, Marusic I, Chong MS (2009). Hot-wire spatial resolution issues in wall-bounded turbulence. J Fluid Mech.

[CR33] Iyengar AKS, Farell C (2001). Experimental issues in atmospheric boundary layer simulations: roughness length and integral length scale determination. J Wind Eng Ind Aerodyn.

[CR34] Jackson PS (1981). On the displacement height in the logarithmic velocity profile. J Fluid Mech.

[CR35] Jimenez J (2004). Turbulent flows over rough walls. Annu Rev Fluid Mech.

[CR36] Kanda M, Moriwaki R, Kasamatsu F (2004). Large-eddy simulation of turbulent organized structures within and above explicitly resolved cube arrays. Boundary-Layer Meteorol.

[CR37] Keirsbulck L, Labraga L, Mazouz A, Tournier C (2002). Surface roughness effects on turbulent boundary layer structures. J Fluid Eng.

[CR38] Krogstad PA, Antonia RA (1999). Surface roughness effects in turbulent boundary layers. Exp Fluids.

[CR39] Krogstad PA, Efros V (2010). Rough wall skin friction measurements using a high resolution surface balance. Int J Heat Fluid Flow.

[CR40] Krogstad PA, Antonia RA, Browne LW (1992). Comparison between rough and smooth wall turbulent boundary layers. J Fluid Mech.

[CR41] Lee SH, Kim JH, Sung HJ, Nickels TB (2010). Direct numerical simulation and PIV measurement of turbulent boundary layer over a rod-roughened wall. IUTAM symposium on the physics of wall-bounded turbulent flows on rough walls.

[CR42] Leonardi S, Castro IP (2010). Channel flow over large cube roughness: a direct numerical simulation study. J Fluid Mech.

[CR43] Leonardi S, Orlandi P, Smalley RJ, Djenidi L, Antonia RA (2003). Direct numerical simulations of turbulent channel flow with transverse square bars on one wall. J Fluid Mech.

[CR44] Ligrani PM, Moffat RJ (1986). Structure of transitionally rough and fully rough turbulent boundary layers. J Fluid Mech.

[CR45] Macdonald RW (1998). An improved method for the estimation of surface roughness of obstacle arrays. Boundary-Layer Meteorol.

[CR46] Mejia-Alvarez R, Christensen KT (2013). Wall-parallel stereo particle-image velocimetry measurements in the roughness sublayer of turbulent flow overlying highly irregular roughness. Phys Fluids.

[CR47] Millward-Hopkins JT, Tomlin AS, Ma L, Ingham D, Pourkashanian M (2011). Estimating aerodynamic parameters of urban-like surfaces with heterogeneous building heights. Boundary-Layer Meteorol.

[CR48] Moody LF (1944). Friction factors for pipe flow. Trans ASME.

[CR49] Napoli E, Armenio V, DeMarchis M (2008). The effect of the slope of irregularly distributed roughness elements on turbulent wall-bounded flows. J Fluid Mech.

[CR50] Nikora V, McLean S, Coleman S, Pokrajac D, Walters R (2007). Double-averaging concept for rough-bed open-channel and overland flows: theoretical background. J Hydraul Eng.

[CR51] Nikuradse J (1933) Laws of flow in rough pipes. Tech. Rep. 1292, NACA Tech. Memo

[CR52] Padhra A (2010) Estimating the sensitivity of urban surface drag to building morphology. Ph.D. thesis, University of Reading, Department of Meteorology, University of Reading

[CR53] Pearson DS, Goulart PJ, Ganapathisubramani B (2013). Turbulent separation upstream of a forward-facing step. J Fluid Mech.

[CR54] Perry AE, Abell CJ (1977). Asymptotic similarity of turbulence structures in smooth- and rough-walled pipes. J Fluid Mech.

[CR55] Perry AE, Li J (1990). Experimental support for the attached-eddy hypothesis in zero-pressure-gradient turbulent boundary layers. J Fluid Mech.

[CR56] Placidi M (2015) On the effect of surface morphology on wall turbulence. Ph.D. thesis, University of Southampton. Engineering and the Environment, University of Southampton

[CR57] Placidi M, Ganapathisubramani B (2015). Effects of large roughness on aerodynamic parameters and the roughness sublayer in turbulent boundary layers. J Fluid Mech.

[CR58] Raupach MR (1992). Drag and drag partition on rough surfaces. Boundary-Layer Meteorol.

[CR59] Raupach MR, Shaw RH (1982). Averaging procedures for flow within vegetation canopies. Boundary-Layer Meteorol.

[CR60] Reynolds RT, Castro IP (2008). Measurements in an urban-type boundary layer. Exp Fluids.

[CR61] Santiago JL, Coceal O, Martilli A, Belcher SE (2008). Variation of the sectional drag coefficient of a group of buildings with packing density. Boundary-Layer Meteorol.

[CR62] Schlichting H (1937) Experimental investigation of the problem of surface roughness. Tech. Rep. 823, NACA Tech. Memo

[CR63] Schlichting H (1979). Boundary layer theory.

[CR64] Schultz MP, Flack KA (2005). Outer layer similarity in fully rough turbulent boundary layers. Exp Fluids.

[CR65] Schultz MP, Myers A (2003). Comparison of three roughness function determination methods. Exp Fluids.

[CR66] Segalini A, Örlü R, Alfredsson HP (2013). Uncertainty analysis of the von Kármán constant. Exp Fluids.

[CR67] Squire DT, Morrill-Winter C, Hutchins N, Schultz MP, Klewicki JC, Marusic I (2016). Comparison of turbulent boundary layers over smooth and rough surfaces up to high Reynolds numbers. J Fluid Mech.

[CR68] Tachie MF, Bergstrom DJ, Balachandar R (2004). Roughness effects on a mixing properties in open channel turbulent boundary layers. Exp Fluids.

[CR69] Townsend AA (1976). The structure of turbulent shear flow.

[CR70] Vanderwel C, Ganapathisubramani B (2015). Effects of spanwise spacing on large-scale secondary flows in rough-wall turbulent boundary layers. J Fluid Mech.

[CR71] Volino RJ, Schultz MP, Flack KA (2007). Turbulence structure in rough- and smooth-wall boundary layers. J Fluid Mech.

[CR72] Volino RJ, Schultz MP, Flack KA (2009). Turbulence structure in a boundary layer with two-dimensional roughness. J Fluid Mech.

[CR73] Volino RJ, Schultz MP, Flack KA (2011). Turbulence structure in boundary layers over periodic two- and three-dimensional roughness. J Fluid Mech.

[CR74] Wu Y (2014). A study of energetic large-scale structures in turbulent boundary layer. Phys Fluids.

[CR75] Wu Y, Christensen KT (2006). Population trends of spanwise vortices in wall turbulence. J Fluid Mech.

[CR76] Wu Y, Christensen KT (2007). Outer-layer similarity in the presence of a practical rough-wall topography. Phys Fluids.

[CR77] Wu Y, Christensen KT (2010). Spatial structure of a turbulent boundary layer with irregular surface roughness. J Fluid Mech.

[CR78] Yang XIA (2016). On the mean flow behaviour in the presence of regional-scale surface roughness heterogeneity. Boundary-Layer Meteorol.

[CR79] Yang XIA, Meneveau C (2015). Recycling inflow method for simulations of spatially evolving turbulent boundary layers over rough surfaces. J Turbul.

[CR80] Yang XIA, Sadique J, Mittal R, Meneveau C (2016). Exponential roughness layer and analytical model for turbulent boundary layer flow over rectangular-prism roughness elements. J Fluid Mech.

[CR81] Yuan J, Piomelli U (2014). Estimation and prediction of the roughness function on realistic surfaces. J Turbul.

[CR82] Zhu X, Lungo VG, Leonardi S, Anderson W (2016). Parametric study of urban-like topographic statistical moments relevant to a priori modelling of bulk aerodynamic parameters. Boundary-Layer Meteorol.

